# Ultrasound features of multinodular goiter in DICER1 syndrome

**DOI:** 10.1038/s41598-022-19709-0

**Published:** 2022-09-23

**Authors:** Marek Niedziela, Karl Muchantef, William D. Foulkes

**Affiliations:** 1 Institute of Pediatrics, Department of Pediatric Endocrinology and Rheumatology, Karol Jonscher’s Clinical Hospital, Poznan University of Medical Sciences, 27/33 Szpitalna Street, 60-572 Poznan, Poland; 2 Department of Radiology, Montreal Children’s Hospital, McGill University Health Centre, Montreal, QC Canada; 3 Department of Human Genetics, McGill University, Montreal, Canada; 4 Department of Medical Genetics, McGill University Health Centre, Montreal, QC Canada; 5 Lady Davis Institute, Jewish General Hospital, Montreal, QC Canada

**Keywords:** Endocrinology, Head and neck cancer, Thyroid diseases, Clinical genetics

## Abstract

DICER1 syndrome is caused by germline pathogenic mutations in the *DICER1* gene. Multinodular goiter (MNG) is a common clinical feature of DICER1 syndrome in children and adults. The aim of this study was to determine the ultrasound (US) characteristics of MNG in patients with DICER1 syndrome. This retrospective study evaluated thyroid US in patients with *DICER1* germline mutations (*DICER1*mut+) performed between 2011 and 2018 at a single center by the same pediatric endocrinologist, and the images were re-examined by an independent pediatric radiologist from another academic center. Patients < 18 years with *DICER1*mut+ and *DICER1*mut+ parents without previous thyroidectomy were included. Ultrasound phenotypes of MNG in the setting of *DICER1* mutations were compared with known US features of thyroid malignancy. Thirteen *DICER1*mut+ patients were identified (10 children, 3 adults). Three children had a normal thyroid US; therefore, thyroid abnormalities were assessed in seven children and three adults. In both children and adults, multiple (≥ 3) mixed (cystic/solid) nodules predominated with single cystic, single cystic septated and single solid nodules, occasionally with a “spoke-like” presentation. All solid lesions were isoechogenic, and in only one with multiple solid nodules, intranodular blood flow on power/color Doppler was observed. Remarkably, macrocalcifications were present in all three adults. The spectrum of ultrasonographic findings of MNG in *DICER1*mut+ patients is characteristic and largely distinct from typical features of thyroid malignancy and therefore should inform physicians performing thyroid US of the possible presence of underlying DICER1 syndrome.

## Introduction

DICER1 syndrome (OMIM#601200) is a familial cancer predisposition syndrome caused by pathogenic germline variants (mutations) in *DICER1.* This gene encodes DICER1, which is a member of the ribonuclease III family of proteins and is involved in the generation of microRNAs, which modulate gene expression at the posttranscriptional level^1^. The spectrum of neoplasms associated with *DICER1* germline pathogenic variants mostly occurs early in life and involves the lungs (pleuropulmonary blastoma), kidneys (cystic nephroma, anaplastic sarcoma of the kidney), female genitourinary system (ovarian sex cord-stromal tumors and embryonal rhabdomyosarcoma of the ovary/bladder/cervix), thyroid (nodular hyperplasia and differentiated thyroid cancer), and eye and brain (ciliary body medulloepithelioma (dictyoma), pineoblastoma and pituitary blastoma)^1^.

Early-onset or familial MNG should prompt a careful personal and family history focused on *DICER1*-associated tumors. The identification of mutations in *DICER1* at the locus MNG1 (previously linked to 14q) in persons with MNG^2^ was the starting point of this study. These data and the paper of Rath et al.^3^ alerted endocrinologists and radiologists to the possibility of DICER1 syndrome in patients in whom childhood MNG is detected. Khan et al*.*^4^ quantified for the first time the excess risk of MNG and thyroid cancer in a cohort of *DICER1* mutation carriers (heterozygotes). Their data supported a significantly increased risk of MNG compared with family controls and a significantly elevated risk of thyroid cancer compared with the population data from the NCI-SEER program. Their data also showed that three of four women and one of six men with DICER1 syndrome will develop MNG or undergo thyroidectomy by age 40 years. Moreover, in those undergoing partial thyroidectomy, most will develop MNG in residual thyroid tissues or will require additional thyroid surgery. Clinically, nodular thyroid disease might be the most common phenotypic abnormality in individuals with germline DICER1 mutations (in particular, in females), with very high penetrance.

MNG seems to be the most common clinical feature of DICER1 syndrome in children and adolescents^4,5^; however, it may also occur in other hereditary tumor syndromes, such as inherited medullary thyroid carcinoma, inherited papillary thyroid carcinoma, intestinal polyposis syndromes, *PTEN* hamartoma tumor syndrome, Carney complex type or Werner’s syndrome^6^ (Suppl. Table 1).

The benefit of radiographic screening is greater for diseases with a higher screening yield^7^, and conversely, syndromes such as DICER1 syndrome will be more efficiently diagnosed if radiologists and endocrinologists select suitable patients for further genetic testing.

To date, little is known about the ultrasonographic phenotype of MNG in DICER1 syndrome. Moreover, it is unknown whether nodular thyroid disease in DICER1 syndrome is an obligatory precursor of differentiated thyroid cancer (DTC), although we and others have shown that DTC is part of DICER1 syndrome^8–10^. In this study, we aimed to describe the US characteristics of MNG in patients with DICER1 syndrome. The thyroid ultrasound (US) features of MNG in the setting of DICER1 syndrome have not yet been widely reported.

## Materials and methods

This retrospective study evaluated thyroid ultrasound studies in patients with *DICER1* germline mutations performed between 2011 and 2018 in a single academic center. Patients ≤ 18 years with *DICER1* germline mutations and an intact thyroid gland were identified and included. Mutation-positive parents without previous thyroidectomy were also included. All patients were examined by the same pediatric endocrinologist with > 25 years of experience performing thyroid ultrasound. All images were subsequently re-examined in 2018 by an experienced pediatric radiologist from another academic center (McGill University Health Centre, Montreal, QC, Canada). Images were assessed by consensus. Thyroid ultrasound was performed with Aloka SSD 3500 (up to March 2016) and Toshiba Premium Aplio 400 (following March 2016). Images were analyzed according to the recent classification of Bueno et al*.*^11^, who proposed four imaging categories of lesions (I—simple cyst, Is—septated cyst, II—mixed cystic and solid, III—solid). Radiologists are often the first practitioners to observe these diverse manifestations and thus play a primary role in recognizing DICER1 syndrome^12^.

US features of thyroid malignancy such as a solitary solid lesion, hypoechogenicity, subcapsular localization with thyroid capsule deformation and invasion, irregular margins, invasive growth (no compression of adjacent tissues), microcalcifications (< 2 mm; found mainly in papillary and medullary thyroid carcinoma), hypervascularity (with normal TSH), suspicious regional lymph nodes, and shape of the lesion “taller than wider” were considered in each examined patient^6,13,14^.

All pathogenic variants in *DICER1* (referred to hereon as *DICER1*mut+) were identified in a single research laboratory at McGill University, and all were confirmed using orthogonal molecular techniques.

This study was performed in accordance with the Helsinki Declaration and good clinical practice and approved by the Institutional Review Board of the Faculty of Medicine of McGill University, Montreal, QC, Canada (Nos. A12-M117-11A and A08-M61-09B) and the Bioethical Commission on Human Research, Poznan University of Medical Sciences, Poznan, Poland (Nos. 257/01 and 258/01). Participants were recruited to the study in compliance with the second edition of the Canadian Tri-Council Policy Statement of Ethical Conduct of Research Involving Humans. All adult participants and the parents of minor subjects gave their written informed consent to participate in the study and to publish the cases (including any images).

## Results

### Patient cohort

US images of ten persons (7 children and 3 adults) diagnosed with MNG between 2011 and 2018 were reviewed. All patients were clinically and hormonally euthyroid with normal free thyroid hormones and TSH levels. We examined 174 static ultrasound images of *DICER1*mut+ patients in total (128 in children and 46 in adults). These 174 images were exclusive to patients in this study, *DICER1*mut+ .

The clinical features of and imaging findings from these ten persons are described in Table 1. The age at presentation of MNG in children and adolescents was between 6 and 16 years. The patient history and clinical and imaging characteristics of two sample patients (< 18 years) recruited before *DICER1* was identified as a tumor susceptibility gene are shown in Suppl. Table 2 and Suppl. Fig. 1 for completion.

**Table 1:** The history, clinical, histopathological and imaging characteristics of multinodular goiter (MNG) patients.

Patient	Age at diagnosis (years)	Sex F female M male	Indication for DICER1 genetic testing	3 or more lesions	Imaging morphological classification of MNG (I, Is, II, III)	Histopathology of MNG	Other clinical features of DICER1 syndrome (at diagnosis)	References
1	14	F	Clinical	Yes	II > I	Nodular goiter	SLCT (6 years)	Sabbaghian et al.^22^
2	16	F	Clinical	Yes	II > Is > III	Follicular adenoma Colloid nodular goiter	cERMS (14 years)	Not previously published
3	7	F	Clinical	Yes	II > III	Nodular colloid goiter with pseudopapillary areas	Not present	Not previously published
4	15	F	Clinical	Yes	III > II	PTCvF/NIFTP? Colloid and hyperplastic nodules	Lung cyst (16 years)	van der Tuin et al.^10^
5	13	M	Clinical	Yes	II > Is > III	Nodular goiter	Not present	Not previously published
6	8	F	Family history and clinical	Yes	II > Is > III	Nodular colloid goiter	Not present	Not previously published
7	14	F	Clinical	Yes	II > III > Is > I	Colloid and hyperplastic nodules	Not present	Not previously published yet
8	23	M	Family history and clinical	Yes	II > III > I macrocalcifications	Not operated yet	Not present	Not previously published
9	30	M	Family history and clinical	Yes	II > III > I macrocalcifications	Not operated yet	Not present	Not previously published yet
10	37	F	Family history and clinical	Yes	II > III > I > Is macrocalcifications	Not operated yet	Not present	Not previously published

*cERMS* cervical embryonal rhabdomyosarcoma, *SLCT* Sertoli-Leydig cell tumour.

**Figure 1:**
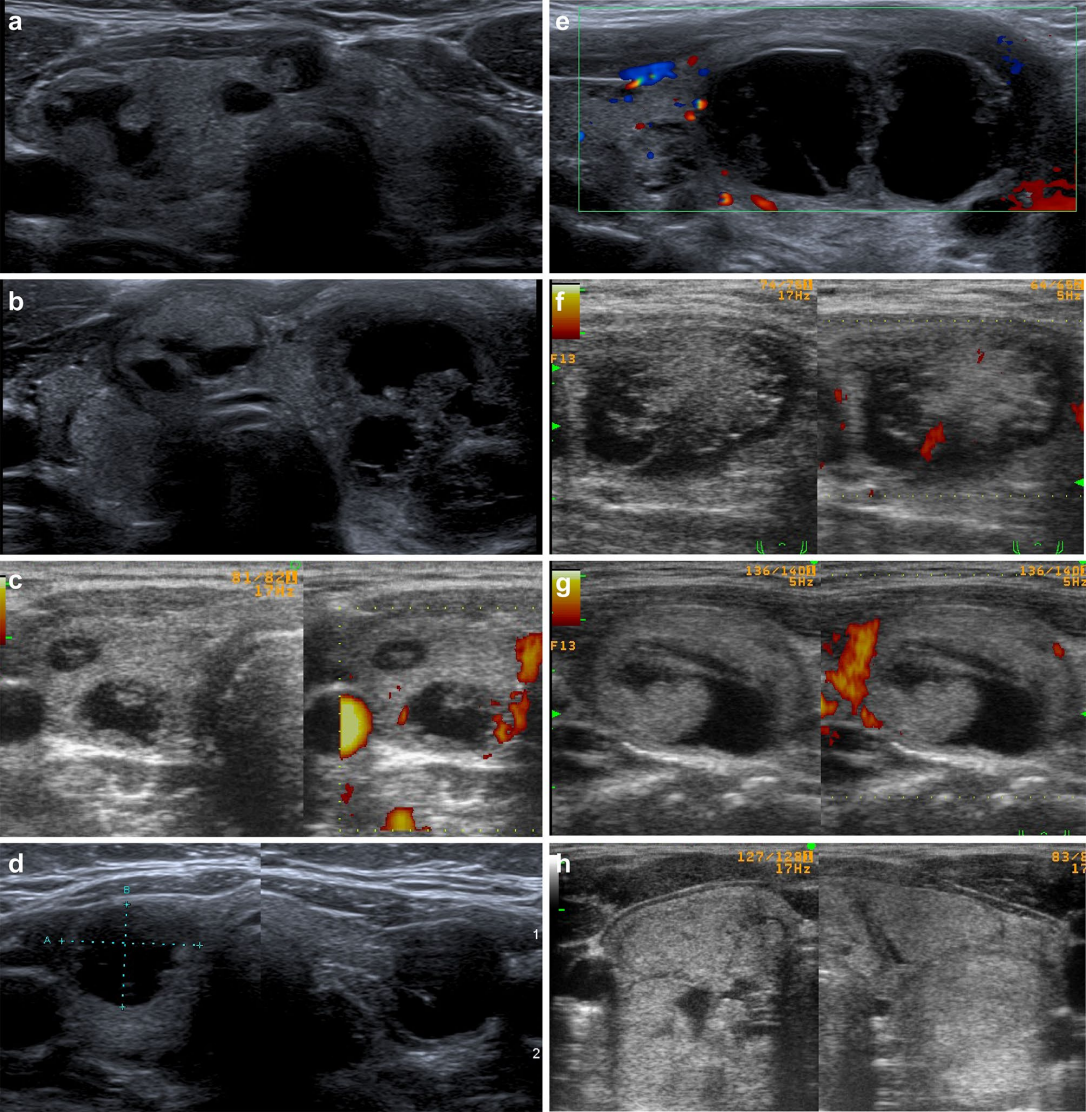
**(a-h)** Ultrasonographic presentation of MNG in all *DICER1*mut+ pediatric patients. (**a–c**) A classic image of MNG for DICER1 syndrome—multiple focal lesions within thyroid (“polymorphic mix”) with dominating mixed cystic and solid nodules (type II) and single cystic (type I) or solid (type II) lesions; (**d**) type I (simple cysts); (**e**) type Is (septated cyst); (**f,g**) type II (mixed cystic and solid); (**h**) type III (solid).

**Figure 2:**
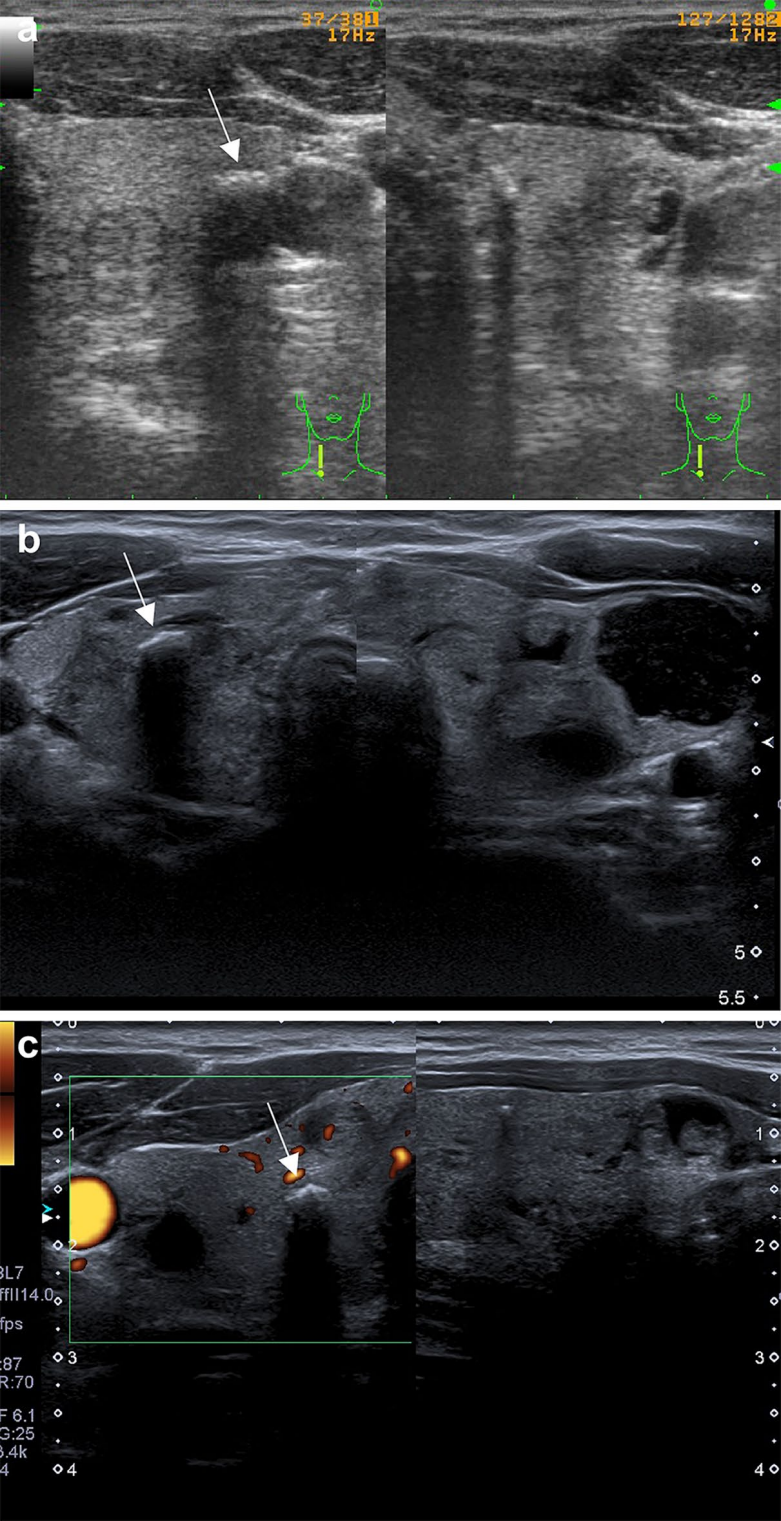
**(a–c)** Ultrasonographic presentation of MNG in *DICER1*mut+ adult patients. A classic image of MNG for DICER1 syndrome; macrocalcifications were also observed (marked with white arrows).

In both adult and pediatric patients, mixed cystic and solid nodules predominated (type II). One adolescent girl had almost all solid isoechogenic nodules, except one hypoechogenic solid area in one nodule, and this patient also had one mixed cystic and solid nodule. Single simple cysts, septated cysts and small solid lesions were detected in all patients without any significant pattern. The patients’ data are summarized in Table 1, and the US presentation of MNG in *DICER1*mut+ pediatric patients is shown in Fig. 1a–h. Ultrasonographic presentation of MNG in *DICER1*mut+ adult patients is shown in Fig. 2a–c.

**Figure 3:**
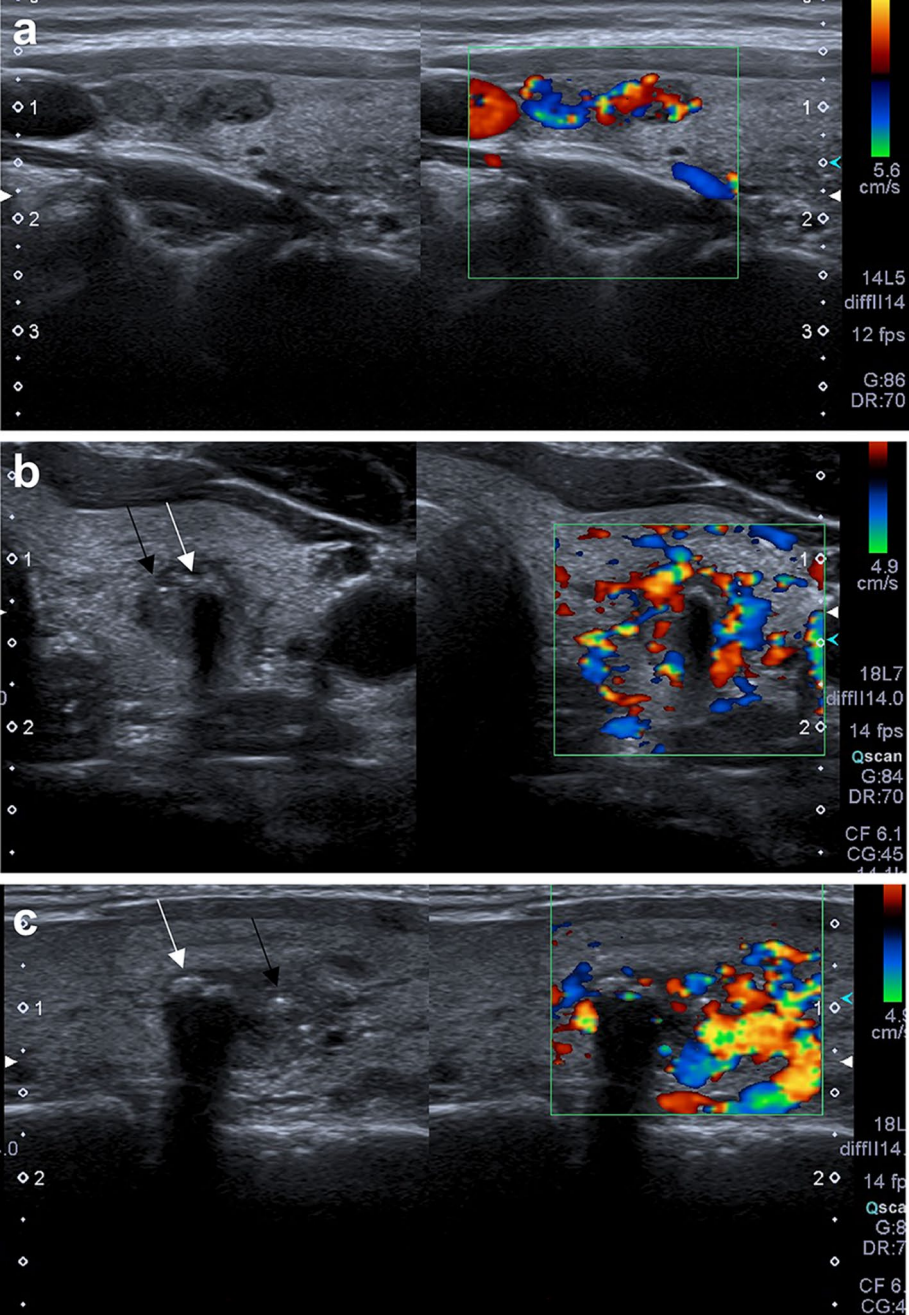
**(a–c)** Ultrasonographic presentation of familial MNG in *DICER1*mut- patients. (**a**) The proband with neuroblastoma and MNG but solid hypoechogenic lesions with increased blood flow on Color Doppler. The histopathological result was hyperplastic nodules/nodular goiter. (**b,c**) The proband’s mother with MNG, cystic and solid lesions with increased blood flow on color Doppler. Both macrocalcifications (white arrows) and microcalcifications (black arrows) were observed. The patient has not yet undergone surgery.

In Fig. 3a–c, we present the ultrasonographic presentation of familial MNG in a patient (after treatment for neuroblastoma) and her mother, both of whom were negative for the *DICER1* germline mutation. Thyroid lesions in this patient were solid, hypoechogenic and showed increased blood flow on color Doppler (Fig. 3a), in contrast to the vast majority of *DICER1*mut+ patients for whom blood flow was absent (except patient 4, see Table 1). Her mother also had multiple thyroid lesions with similar solid hypoechogenic lesions and increased blood flow on color Doppler; additionally, she had macrocalcifications, as in adult *DICER1*mut+ patients, and microcalcifactions, which are frequently observed in PTC. Thyroid nodules in one *DICER1*mut+ case with a PTCvF microcarcinoma (patient 4, see Table 1), and in a girl with neuroblastoma (NB) and her mother showed increased vascular flow in the CD/PD, but these patients also had normal levels of TSH and free thyroid hormones.

**Figure 4:**
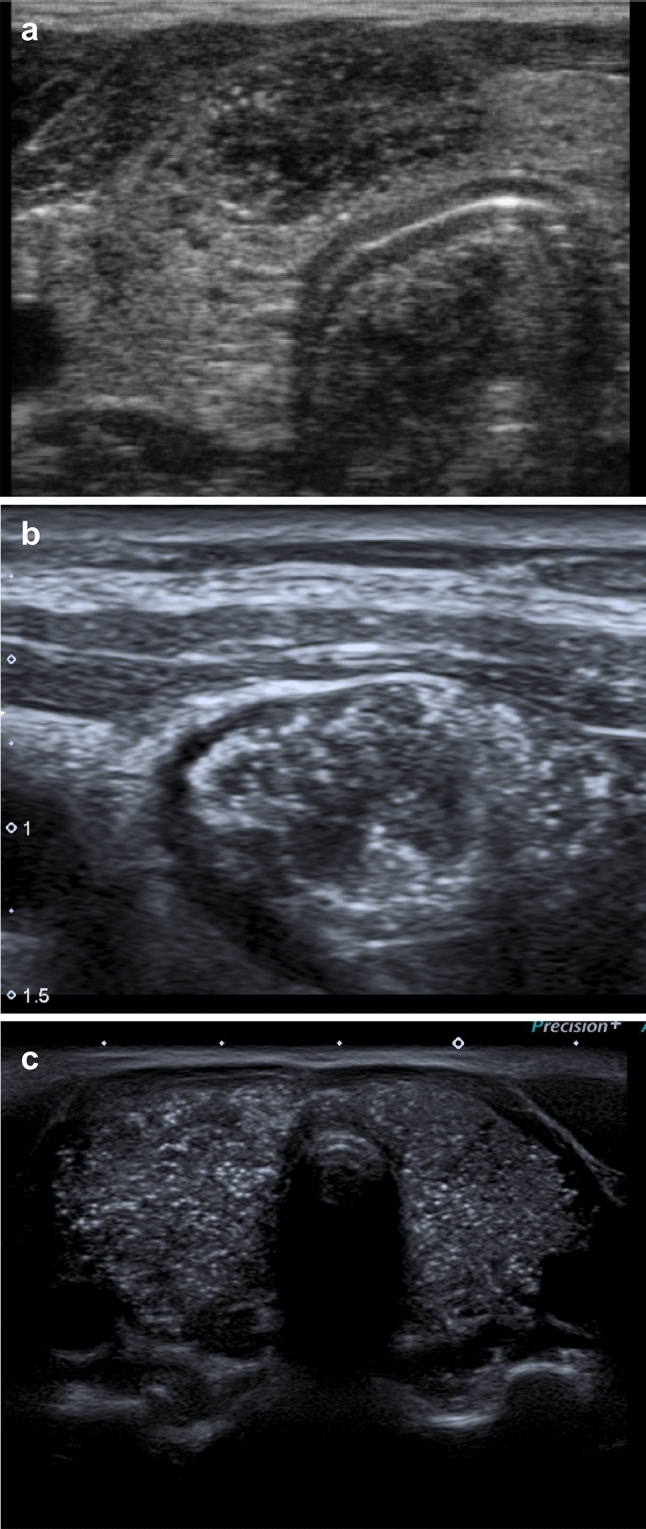
**(a–c)** Ultrasonographic presentation of papillary thyroid carcinoma. (**a**) A solitary hypoechogenic nodule with subcapsular localization, irregular borders and multiple microcalcifications in an adolescent boy with coexisting autoimmune thyroiditis. (**b**) A solitary hypoechogenic nodule with subcapsular localization and multiple microcalcifications in an adolescent girl with coexisting Graves’ disease. (**c**) Multiple disseminated microcalcifications in an adolescent girl with hypoechogenic thyroid gland and coexisting autoimmune thyroiditis (multifocal diffuse sclerosing PTC).

Ultrasonographic phenotypes of MNG of *DICER1*mut+ patients were also compared with known ultrasonographic features of thyroid malignancy, and the results are shown in Fig. 4a–c. This comparison, summarized in Tables 2 and 3, clearly presents the differences in imaging between the two groups of patients. Briefly, *DICER1*mut+ patients lack classic ultrasonographic features of thyroid malignancy, such as a solitary nodule, hypoechogenicity, irregular margins of the lesion, increased blood flow, the presence of microcalcifications with a taller than wider shape, and accompanying “suspicious” lymph node/s.

**Table 2:** Ultrasonographic features of thyroid malignancy and their presence in MNG in patients with DICER1 syndrome (n = 10; 7 < 18 years and 3 ≥ 18 years).

	Thyroid cancer	MNG/DICER1 (number of patients)
1. Solitary solid lesion	+	0/10
2. Hypoechogenic	+	0/10
3. Subcapsular localization with thyroid capsule invasion	+	0/10
4. Irregular margins	+	0/10
5. Invasive growth (no compression of adjacent tissues)	+	0/10
6. Microcalcifications (< 2 mm; found mainly in PTC and MTC)	+	0/10
7. High intranodular flow by Doppler (with normal TSH)	+	1/10
8. Suspicious regional lymph nodes accompanying thyroid nodule	+	0/10
9. Shape of the lesion: “taller” than “wider”	+	0/10
10. Firm (no plasticity) on elastosonography	+	Not evaluated

*PTC* papillary thyroid carcinoma, *MTC* medullary thyroid carcinoma.

**Table 3:** Characteristic features of MNG in DICER1 syndrome and their presence in thyroid cancer (n = 10; 7 < 18 years and 3 ≥ 18 years).

	MNG/DICER1 (number of patients)	Thyroid cancer
1. Multiple lesions (≥ 3)	10/10	Infrequent but may happen
2. Predominantly mixed solid and cystic nodules (type II)	9/10	Infrequent but may happen
3. Spoke-like lesions (< 18 years); n = 7	2/7	Rarely present
4. Lack of blood flow or exclusively in solid nodules	9/10	Infrequent but may happen
5. Lack of suspicious lymph nodes on the neck	10/10	Infrequent but may happen
6. Macrocalcifications (≥ 18 years); n = 3	3/3	Infrequent but may happen

As yet, we have not found a way to determine whether these ultrasound findings are truly specific for, and characteristic of, DICER1 syndrome-related MNG. There are precious few publications on ultrasound features of pediatric MNG, and comparative studies are lacking, so we have relied upon the subjective experience of M.N, who has for 26 years being conducting pediatric thyroid ultrasounds. It is on this basis that we consider *DICER1*mut+ lesions to be distinct from other forms of multiple benign thyroid lesions.

We did not find an association of the *DICER1* mutation with a specific ultrasound image and we also did not find an association of the *DICER1* mutation with progression to PTCvF.

All patients in this study manifested MNG at time of diagnosis. There was no relationship between the age of MNG diagnosis and any molecular diagnosis. Long-term prospective follow-up of patients with DICER1 syndrome who remain thyroid disease-free is ongoing and may shed a new light on the pathogenesis of *DICER1*-related MNG.

## Discussion

To our knowledge, this is the first survey of thyroid ultrasound characteristics conducted in patients with MNG in DICER1 syndrome who were all confirmed by genetic testing to have a pathologic germline *DICER1* mutation. We have presented the ultrasonographic features of MNG in DICER1 syndrome in descriptive terms. We took this approach because the ideal control group (children with MNG, who do not have germline DICER1 mutations) is not available and no publications exist for retrospective comparison. Indeed, it is likely that *DICER1* is the main or only genetic cause of pure MNG in childhood, so it is difficult to compare DICER1 to other extremely rare syndromes, such as *PTEN* hamartoma tumor syndrome. Some studies have suggested that neuroblastoma (NB) may also be involved in this syndrome. Saskin et al.^15^ described the case of a 14-year-old female presenting with a multinodular goiter (MNG) and a collision tumor composed of NB and cystic nephroma (CN). She carried a deleterious germline mutation in exon 23 of *DICER1* and harbored different somatic mutations in the CN and MNG. However, no second hit was found in the NB, leading to its status as a DICER1-related tumor being questioned. We have shown in Fig. 3a–c that such a family with MNG and the proband had a prior NB, but a *DICER1* germline mutation was not present in this family.

The only one DICER1mut+ patient diagnosed in our center with thyroid microcarcinoma (PTCvF) had no classical ultrasound features of malignancy, but we cannot exclude that the other patients worldwide diagnosed with thyroid cancer may manifest such features.

Large cohorts, particularly those prospectively studied, may provide an answer to questions such as (a) at what age can thyroid nodular disease start in DICER1 syndrome; (b) how dynamic is the process; and (c) does it progress toward cancer regardless of whether it is initially benign or malignant, or do potential triggering factors need to be present? Larger patient series may allow for statistical analyses and validation of our findings in the future.

The scant data on DTC in DICER1 syndrome may lead to questions regarding whether and when these patients should undergo thyroidectomy; moreover, what about the extent of surgery (total, near/total or not at all) if they still are euthyroid, have benign cytology from fine needle aspiration (category 2 based on Bethesda system)^16^, do not have symptoms from their goiter, or there is a very low risk for cancer? The most recent data on DICER1 syndrome in adolescent DTC suggest that germline mutations in *DICER1* are more likely to lead to MNG than to DTC and that if DTC does occur in the context of biallelic *DICER1* mutations, it is likely to be a low-risk tumor^17^. In a registry-based study, Khan et al*.*^4^ found that *DICER1* carriers have a statistically significant, 16- to 18-fold increased risk of developing DTC and that this risk is increased to 24-fold after censoring of complete and partial thyroidectomies.

Among young individuals operated for benign MNG (nodular goitre, colloid nodular goitre, and hyperplastic nodular goitre) below age 25 years, 13% were found to possess a germline pathogenic variant in DICER1^18^.

In two reports^4,17^ the authors stated that *DICER1*-associated thyroid cancer is not more invasive or less responsive to therapy than its non-*DICER1*-associated counterpart, which is supported by a more recent study^17^.

*DICER1* mutations in pediatric PTC are present at a frequency nearly 30 times that seen in adult PTC. Sequencing of *DICER1* identified pathogenic somatic variants in 10% PTCs, all of which lacked conventional alterations. Germline *DICER1* pathogenic variants were identified in 20% of benign lesions. These data establish *DICER1* as a common oncogenic driver in American Thyroid Association pediatric low-risk PTC and broaden our understanding of the molecular pathogenesis of pediatric PTCs^17^.

We agree with Khan and coworkers^4^ that *DICER1* mutation carriers with a thyroid nodule should receive standard management before consideration for thyroid resection, including thyroid and neck ultrasonography, to assess evidence of bilateral thyroid disease and metastasis to cervical lymph nodes^19,20^. However, in our experience, these patients do not usually present with a solitary thyroid nodule but instead have at least three nodules; therefore, the diagnostic and therapeutic protocol should differ from that for solitary nodules. In our series, the decision whether to operate or not was based on clinical data, i.e., the presence of a large multinodular goiter filling almost the entire volume of the gland. A fine needle biopsy ruled out thyroid cancer, although with multinodular lesions there is still uncertainty because not all nodules in these children are biopsied in such cases. At present, the literature indicates that the risk of cancer in children and adolescents is low, but preoperative biopsy does not detect all thyroid cancers. Another facet is that in the context of DICER1 syndrome, it is extremely likely that a partial thyroidectomy will need to be followed by a completion thyroidectomy at some point. In addition, childhood- and adolescent-onset poorly differentiated thyroid carcinoma (PDTC) has been shown to be associated with *DICER1* mutations and may herald DICER1 syndrome in some patients. Furthermore, their clinically aggressive behavior contrasts sharply with the indolent nature of the great majority of thyroid tumors with *DICER1* mutations reported to date^21^. Thus, while most lesions will follow a benign course, total thyroidectomy is the procedure of choice for the above-mentioned reasons.

Total thyroidectomy as the procedure of choice is recommended for the above-mentioned reason, since it avoids further glandular proliferation of an already significantly enlarged gland with features of multinodular goiter and nodular disease recurrence in the case of partial thyroidectomy and allows to diagnose cancer at early stage.

Clinical and ultrasonographic follow-up on 6-month intervals or at least on annual basis is mandatory in non-operated patients with DICER1-related MNG in order to detect early a rare but potentially possible transformation into the suspicious lesion that need biopsy and more radical treatment.

In terms of management of other mendelian forms of MNG, a retrospective review of prospectively accrued PTEN hamartoma tumor syndrome (PHTS) patients suggests stratifying surveillance intervals based on thyroid ultrasound result, and support extending surveillance intervals in PHTS patients without nodules on ultrasound to 3–5 years, and patients with clinically nonactionable nodules to 2–3 years, which is in contrast to the current recommendation of annual ultrasounds. This change in practice would decrease the burden of frequent ultrasounds, especially in young children and adolescents that are more likely to have a normal or nonactionable ultrasound result^24^.

Long-term prospective follow-up of patients with DICER1 syndrome who remain thyroid disease-free is ongoing and may shed a new light on active surveillance in *DICER1*mut+ patients to propose the most accurate frequency of control ultrasound examinations.

Clinicians should consider in the diagnostic workup that other hereditary disorders/syndromes (Suppl. Table 1) may also manifest with multinodular goiter, but based on our experience, nodules in patients with DICER1 syndrome, particularly those with benign histopathology and those presented in this paper, have a unique and distinguishable pattern. Other etiologies may contribute in MNG pathogenesis, however genetic causes of euthyroid MNG in childhood are all likely to be much less prevalent than DICER1 syndrome.

Euthyroid MNG before 30 years strongly suggests a hereditary cause. DICER1 is in our opinion the dominant candidate in such patients particularly if the ultrasound features we report here are present. The diagnostic protocol should also consider investigation for DICER1 large deletions, either by MLPA^22^ or as part of a larger NGS panel (including genes in Supplementary Table 1) that is capable of detecting copy number variants.

We hope that our US criteria of MNG in DICER1 syndrome, when compared with a classic ultrasonographic presentation of thyroid cancer, will shed new light on which children and adults with MNG should be considered for *DICER1* genetic testing. These criteria could also be useful in determining how likely it is that malignancy will be present. In this vein, the lack of suspicious cervical lymph nodes seems to be an important and adjuvant ultrasonographic feature in evaluating MNG patients. Fine needle aspiration of thyroid nodules is a superior tool for detecting thyroid cancer to optimize the surgical extent and improve the final outcome. We also agree with Khan and coworkers^4^ that there are no data to support prophylactic thyroidectomy in those with DICER1 syndrome, unlike other hereditary syndromes affecting the thyroid, such as multiple endocrine neoplasia type 2^23^. However, if surgery is being performed as definitive treatment, then total thyroidectomy should be advocated, despite the almost universal absence of US features of thyroid malignancy in *DICER1*-mutated MNGs. There is still no answer to the question of whether adjuvant therapy with low doses of radioiodine is warranted for DICER1 syndrome-associated DTC, but based on the current data, we favor a “wait-and-see” approach with annual clinical, hormonal and ultrasonographic evaluation until long-term data are available. Another related issue is the controversial morphological/histopathological presentation of MNG in DICER1 syndrome, given the tendency to diagnose papillary thyroid carcinoma in borderline cases where papillomatous hyperplasia can mimic papillary thyroid carcinoma. We suggest that historically diagnosed PTC with DICER1-related pathological features of their MNG should be re-examined by pathologists with experience in DICER1-related tissue abnormalities.

## Conclusion

The spectrum of MNG in *DICER1-*mutated patients is largely invariant between patients. The patients presented in this paper showed no classic features of thyroid malignancy on ultrasound. The findings that are characteristic of MNG in DICER1 syndrome are (1) multiple lesions (≥ 3) with a predominantly mixed cystic and solid echostructure (type II) and a spoke-like presentation and (2) macrocalcifications in adults. These nodules do not have increased blood flow in solid parts, and suspicious neck lymph nodes are uncommonly seen. Such criteria should inform physicians performing thyroid ultrasound that lesions with these characteristics are likely to be attributable to germline *DICER1* mutations. The prompt identification of DICER1 syndrome as the underlying cause of MNG may prevent multiple thyroid resections.

## Supplementary Information

Supplementary Figure 1.

Supplementary Table 1.

Supplementary Table 2.
