# Supplementary material for: Ultrasound features of multinodular goiter in DICER1 syndrome
Source: Sci Rep. 2022 Sep 23;12:15888. doi: 10.1038/s41598-022-19709-0 (PMC9508228; doi:10.1038/s41598-022-19709-0)
Supplement: Supplementary file 1 — Supplementary Figure 1. [file 41598_2022_19709_MOESM1_ESM.pptx]

## Slide 1
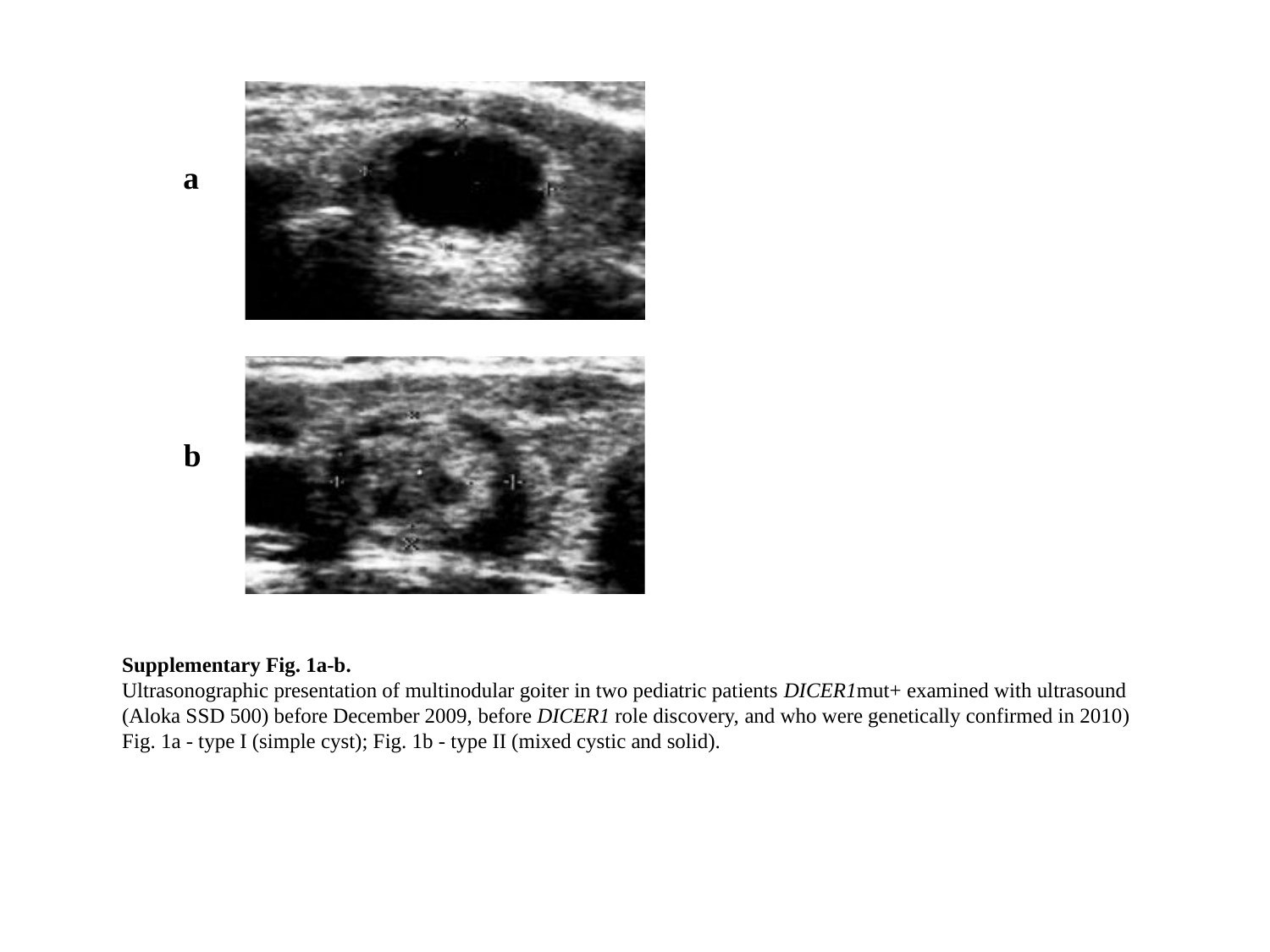

a
b
Supplementary Fig. 1a-b.
Ultrasonographic presentation of multinodular goiter in two pediatric patients DICER1mut+ examined with ultrasound (Aloka SSD 500) before December 2009, before DICER1 role discovery, and who were genetically confirmed in 2010)
Fig. 1a - type I (simple cyst); Fig. 1b - type II (mixed cystic and solid).
