# Supplementary material for: Ultrasound features of multinodular goiter in DICER1 syndrome
Source: Sci Rep. 2022 Sep 23;12:15888. doi: 10.1038/s41598-022-19709-0 (PMC9508228; doi:10.1038/s41598-022-19709-0)
Supplement: Supplementary file 2 — Supplementary Table 1. [file 41598_2022_19709_MOESM2_ESM.docx]

**Supplementary Table 1**. Paediatric hereditary syndromes associated with thyroid cancer. Some of syndromes (DICER1 syndrome,

Carney complex and *PTEN* hamartoma tumor syndrome) may also be associated with benign multinodular goiter.

____________________________________________________________________________________________________________________

Syndrome Gene Locus Mode Thyroid

or familial occurrence neoplasia

_______________________________________________ _____________________________________________________________________

Inherited MTC:

Isolated fMTC *RET* 10q11.2 AD MTC

MEN2A *RET* 10q11.2 AD MTC

MEN2B *RET* 10q11.2 AD MTC

Inherited PTC:

Isolated fPTC ? 2q21 ? PTC

fPTC with or without oxyphilia *TCO/ TIMM44* 19p13.2 AD PTC

fPTC with papillary renal cell carcinoma ? 1q21 ? PTC

fPTC with MNG (DICER1 syndrome) *DICER1* 14q32.13 AD PTC

Pendred syndrome *SLC26A4* 7q31 AR FTC/PTC

Intestinal polyposis syndromes:

Familial adenomatous polyposis (FAP) *APC* 5q21 AD PTC

Peutz-Jeghers syndrome *STK11* *(LKB1)* 19p13.3 AD PTC

*PTEN* hamartoma tumour syndrome (PHTS) *PTEN* 10q23.2 AD FTC/HCC

Carney Complex type 1 *PPRKAR1a* 17q23-24 AD PTC/FTC

2p16

Werner’s syndrome (“adult progeria”) *WRN* 8p11-p12 AR FTC/PTC/ATC

____________________________________________________________________________________________________________________

Abbreviations: MTC – medullary thyroid carcinoma; fMTC – familial medullary thyroid carcinoma; MEN – multiple endocrine neoplasia; fPTC – familial papillary thyroid carcinoma; PTC – papillary thyroid carcinoma; FTC – follicular thyroid carcinoma; MNG – multinodular goiter; HCTC – Hurthle cell thyroid carcinoma; ATC – anaplastic thyroid carcinoma
