# Supplementary material for: Ultrasound features of multinodular goiter in DICER1 syndrome
Source: Sci Rep. 2022 Sep 23;12:15888. doi: 10.1038/s41598-022-19709-0 (PMC9508228; doi:10.1038/s41598-022-19709-0)
Supplement: Supplementary file 3 — Supplementary Table 2. [file 41598_2022_19709_MOESM3_ESM.docx]

**Supplementary Table 2.** Multinodular goiter (MNG) patients’ history, clinical and imaging characteristics of two patients examined with ultrasound (Aloka SSD 500) before December 2009 and genetically confirmed in 2010.

| **Patient** | **Age at diagnosis**  **(years)** | **Sex**  **F female**  **M male** | **Indication**  **for DICER1 genetic testing** | **3 or more lesions** | **Imaging morphological classification of MNG**  **(I, Is, II, III)** | **Histopathology**  **of MNG** | **Other clinical features of**  **DICER1 syndrome**  **(at diagnosis)** | **References** |
| --- | --- | --- | --- | --- | --- | --- | --- | --- |
| **1** | 11 | M | Clinical | Yes | **II**>I>III | Papillomatous nodular hyperplasia.  Hyperplastic nodules. | - | Rio Frio T, et al. [JAMA 2011, 305(1](http://150.254.181.150/scripts/expertus.exe?KAT=c%3A%5Cbib_am%5Cpar%5C&FST=data.fst&FDT=data.fdt&ekran=ISO&lnkmask=2&cond=AND&mask=2&F_00=06&V_00=JAMA+2011+Vol.+305+nr+1)): 68-77. |
| **2** | 9 | F | Clinica; | Yes | **II**>III | Papillomatous nodular hyperplasia.  Hyperplastic nodules. | SLCT (14 yrs)  (Sertoli-Leydig Cell Tumour) | Rio Frio T, et al. [JAMA 2011, 305(1](http://150.254.181.150/scripts/expertus.exe?KAT=c%3A%5Cbib_am%5Cpar%5C&FST=data.fst&FDT=data.fdt&ekran=ISO&lnkmask=2&cond=AND&mask=2&F_00=06&V_00=JAMA+2011+Vol.+305+nr+1)): 68-77. |
